# A systematic review of the use of human body donor models for postgraduate laparoscopic surgical training

**DOI:** 10.1007/s12565-025-00872-z

**Published:** 2025-07-17

**Authors:** Sedat Alp Pinar, Joseph J. Morrow, Chia Yew Kong

**Affiliations:** Academic Unit of Surgery, Glasgow Royal Infirmary and University of Glasgow, New Lister Building, Glasgow, G31 2ER UK

**Keywords:** Cadaver, Education, Laparoscopy, Minimally invasive surgical procedures, Systematic review

## Abstract

While laparoscopy has become the gold standard for many abdominal surgical procedures, it has a steeper learning curve compared to an open surgical approach. Body donor (cadaveric) models (BDM) present a high-fidelity model for laparoscopic surgical training. There are a variety of models available from fresh-frozen models to soft-embalmed models using multiple proprietary preparation solutions. There remains uncertainty surrounding the models’ relative strengths and weaknesses. This study aims to evaluate the different body donor models used in postgraduate laparoscopic surgical training. A systematic search was conducted across Medline, Embase, ClinicalTrials.gov, and Cochrane to identify articles relevant to postgraduate laparoscopic training using HBD following the PRISMA guidelines. A narrative review themed on HBD feasibility, validity, and educational utility in laparoscopic surgical training. PROSPERO Registration: NIHR CRD42023437230. Fifty-five studies were included. BDM in general were perceived as authentic and of high educational value. Soft embalmed models (e.g., Thiel^®^, Genelyn^®^, modified Larssen^®^), were reported to be feasible and possess high face validity despite embalming and had advantages over fresh frozen body donors (FFBDs), including reusability, cost-effectiveness, and better odor satisfaction. Comparative studies reported a preference of human body donor models over other simulation methods due to its high fidelity. Body donor models including soft embalmed models provide high-fidelity laparoscopic training environments well perceived by trainees regardless of preservation or tissue fixation. Significant research gaps remain; head-to-head comparative studies between the different models remain sparse as well as data on non-educational aspects such as formal cost-utility analysis and ecological impact.

**Supplementary Information:**

The online version contains supplementary material available at 10.1007/s12565-025-00872-z.

## Introduction

Laparoscopic surgery is a gold standard approach in a variety of surgical procedures across a range of specialities, most notably general surgery, urology, and gynaecology. Piechaud and Pansadoro (2006); Pattanshetti and Pattanshetti (2010); Stovall et al. (2006); Zevin et al. (2012). Laparoscopy has been shown to be associated with better patient outcomes including shortened length of stay, reduced rate for complications, reduced blood loss. Pattanshetti and Pattanshetti (2010). It is, however, associated with a more difficult learning curve compared to an open approach. Samia et al. (2013).

There has been a shift in surgical training approaches from a traditional apprenticeship model in the operating theatre to a more structured training using different training modalities outside of direct clinical care. This shift in training is not only a result of the more difficult learning curves associated with gaining proficiency in both more traditional open but also laparoscopic approaches, but also the reduced training time to surgical trainees. Samia et al. (2013).

The need for more structured training for laparoscopic surgery has led to the development of a variety of ex vivo models to train surgeons. Pattanshetti and Pattanshetti (2010); Miskovic et al. (2010). These models include body donors, live anaesthetized animal models, bench models and virtual reality trainers. There are multiple factors to be considered when selecting the most appropriate model for training including costs, fidelity, relative effectiveness, availability, and stage of training.

A range of body donor models are widely used in surgical training due to their fidelity. The two primary preparation methods for human body donors used in laparoscopic training are fresh frozen body donors (FFBD), and embalmed body donors using preservatives. Reddy et al. (2017). FFBDs, which is the traditional body donor model for use in surgical training, involves the preservation of body donors by freezing without any embalming. It has well established drawbacks including a short lifespan, high costs associated with the lifespan, odour, and the necessity for refrigeration. Eisma and Wilkinson (2014); Jaung et al. (2011). In contrast, embalmed body donor models for surgical training are traditionally embalmed using high concentrations of formalin, and although these have a longer lifespan, their use in surgical training is limited by their poor tissue flexibility and therefore exclude these models for laparoscopic surgery due to an inability to achieve or maintain gaseous insufflation of the peritoneal cavity necessary for laparoscopy. Lloyd et al. (2011). Developments in embalming techniques have, however, led to the development of soft embalmed models allowing for both a lengthened lifespan and adequate tissue flexibility compared to formalin embalmed body donors. Eisma and Wilkinson (2014); Lloyd et al. (2011). Currently, there are several soft embalming proprietary techniques available, each providing the body donor with different mechanical properties. Jaung et al. (2011).

Despite the large variety of body donor models available there remains no consensus on the fidelity and educational validity of these body donor models for training in laparoscopic surgery and as a proxy simulation model of training in direct clinical care settings.

This systematic review will present the findings looking at the use of different body donor models for laparoscopic surgical training, to discuss whether evidence from current literature suggests the use of a specific body donor model for training over others.

## Materials and methods

**Fig. 1:**
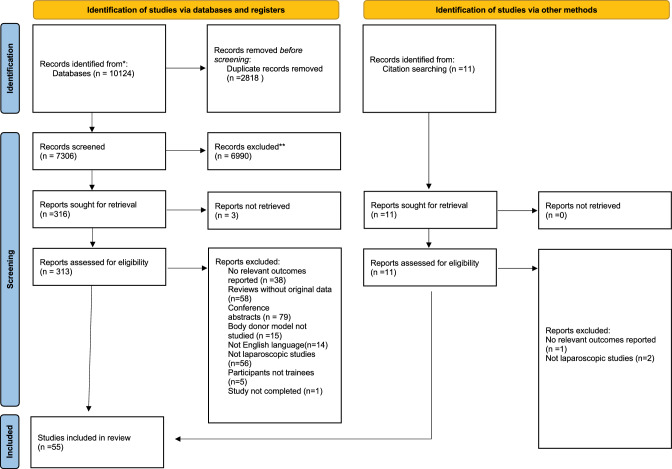
PRISMA flow diagram of the literature search and inclusion process

This systematic review was conducted in accordance with the PRISMA guidelines (Fig. 1) Page et al. (2021). The review protocol was registered with Prospero (NIHR CRD42023437230). Schiavo (2019).

### Search strategy and data sources

A literature search was conducted in July 2023 using MEDLINE (Ovid), EMBASE (Ovid), Clinicaltrials.gov, and Cochrane database. Search results were limited to the English language. Duplicates were removed and this was followed by the screening of retrieved titles and abstracts for eligibility. Reference lists of included studies were also manually searched to achieve adequate literature saturation.

The search strategies for each of the different databases used are presented in Supplement Table 1.

**Table 1:** Four types of validity used to evaluate body donor models in surgical training

Model validity	Definition
Face validity	The extent to which a model accurately resembles a real-life procedure, ensuring authenticity. Rueda Esteban et al. (2023)
Construct validity	The model’s ability to distinguish between participants with varying levels of experience and expertise. Rueda Esteban et al. (2023)
Content validity	The model’s capacity to enhance participants' knowledge and skills in a range of training settings. Rueda Esteban et al. (2023)
Predictive validity	The model’s effectiveness in translating improved knowledge and skills to real-life clinical settings, and in this case, the operating theatre. Gallagher et al. (2003)

Each validity type is defined based on its relevance to training authenticity, differentiation between experience levels, enhancement of knowledge and applicability to real-life clinical settings

### Selection criteria and data extraction

Studies were screened sequentially by two co-authors as with the flowchart for the screening process presented in Fig. 1. Studies were considered eligible if they reported an assessment of educational outcomes of postgraduate doctors on any body donor model as a training model for laparoscopic surgery. Articles were also included if they provided detailed descriptions of the body donor model as part of purely descriptive studies evaluating the feasibility of surgical techniques.

Studies were excluded if animal models were used, involved veterinary trainees or undergraduate medical students, or the operations were done using robotics or natural orifice transluminal endoscopic surgery. Conference abstracts were also excluded.

After eligibility screening, abstracts were retrieved in full text. Reference lists of full-text articles were manually searched, and those identified also had to match the same eligibility criteria.

Two independent reviewers extracted data from full-text articles using piloted extraction forms with any discrepancies resolved by discussion of the two researchers.

Data items collected included body donor training (surgical procedure, body donor model used); participant characteristics (number, specialty, stage in training, surgical experience); assessment of educational impact; and aspects of model validity assessed.

Following data extraction, a narrative review was conducted to synthesize the findings, highlighting key aspects of the body donor models, their educational impact, and their feasibility in surgical training. The findings from the studies and the collected data are presented in the table below.

### Quality assessment

A quality assessment of the articles included in our study through the Modified Medical Education Research Study Quality Instrument (MMERSQI). Al Asmri et al. (2023). In brief, studies are assessed across 6 domains with a maximum score of 3 in each domain and 18 in total. Certain domains are rated as not applicable; where this is the case, scores are scaled to allow comparison of scores between the studies. Two independent reviewers extracted data from full-text articles using pre-designed data extraction forms, with any discrepancies resolved by discussion of the two researchers with the third author.

### Validity criteria

In our literature review, we evaluated the included studies based on their assessment of the validity of body donor models. Rueda Esteban et al. (2023); Gallagher et al. (2003). We categorized validity into four distinct types: face validity, construct validity, content validity, and predictive validity, as outlined in Table 1.

## Results

Figure 1 illustrates the PRISMA flow diagram, detailing our literature search process, including the number of papers screened, retrieved, and excluded, along with the reasons for their exclusion.

The results of our quality assessment are presented in Table 2. 7 studies could not be scored as these were purely descriptive studies. Of the 48 studies we have scored for quality, the scaled scores ranged from 5.4 to 16.4, and no studies achieved a maximum score. The articles included in our study had a median MMERSQI score of 10.2.

**Table 2:** MERSQI scores for articles included in our study

Paper	Study design	Sampling: Institutions	Sampling: response rate	Type of data	Validity evidence for evaluation instrument scores	Data analysis: sophistication	Data analysis: appropriateness	Outcome	Scaled score
Ackermann et al. (2020a)	1	NA	1.5	3	1	2	1	1.5	12.0
Ackermann et al. (2020b)	1	NA	0.5	3	1	2	1	1.5	10.9
AlJamal et al. (2018)	1.5	NA	1.5	3	1	2	1	1.5	12.6
Bilge and Celik (2017)	1	NA	0.5	1	1	NA	0	1	5.4
Blaschko et al. (2007)	1	NA	1.5	1	1	1	0	1	7.1
Britt et al. (2015)	1.5	0.5	1.5	3	1	2	1	1.5	12.0
Cundiff et al. (2001)	2	0.5	1.5	3	1	2	1	1.5	12.5
Danion et al. (2020)	1	1.5	1.5	1	1	2	1	1	10.0
Donatini et al. (2021)	1	NA	NA	1	1	2	1	1	8.4
Foster et al. (2014)	1	NA	1	3	1	1	1	2	10.9
Gamboa et al. (2009)	1	1.5	1.5	3	1	1	1	2	12.0
Giger et al. (2008)	1	1.5	1.5	1	1	2	1	1	10.0
Katz et al. (2003)	2	NA	0.5	1	2	2	1	1	10.4
Leblanc et al. (2010a)	2	NA	1.5	3	2	2	1	1.5	14.2
Leblanc et al. (2010b)	2	NA	1.5	3	2	2	1	1.5	14.2
Levine et al. (2006)	1.5	NA	1.5	3	2	2	1	1.5	13.6
Lewis et al. (2012)	1	0.5	1.5	1	1	1	1	1	8.0
Lim et al. (2018)	2	NA	0.5	1	1	2	1	1	9.3
Mantica et al. (2020)	1	NA	0.5	1	1	2	1	1	8.2
Marcu et al. (2021)	1.5	NA	0.5	3	2	2	1	1.5	12.6
Miller et al. (2019)	2	0.5	1.5	1	1	2	1	1	10.0
Palter and Grantcharov (2012)	3	NA	1	3	2	2	1	3	16.4
Pattana-arun et al. (2005)	1	NA	1.5	1	1	1	1	1	8.2
Rai et al. (2015)	2	NA	1.5	1	2	2	1	1	11.5
Rai et al. (2012)	2	NA	1.5	1	2	2	1	1	11.5
Rashidian et al. (2019)	1	1,5	1	1	1	1	1	1	9.3
Ross et al. (2008)	1	NA	1	3	1	1	1	2	10.9
Ruiz-Tovar et al. (2019)	1	NA	1.5	1	2	2	1	1	10.4
Sanchez-Ferrer et al. (2019)	1	NA	0.5	1	1	1	1	1	7.1
Sanchez-Ferrer et al. (2020)	1	NA	0.5	1	1	1	1	1	7.1
Sharma and Horgan (2012)	2	NA	1.5	1	2	2	1	1	11.5
Sharma et al. (2013)	3	NA	1.5	3	1	2	1	1.5	14.2
Sharma et al. (2012)	3	NA	1.5	3	1	2	1	1.5	14.2
Slieker et al. (2012)	1	NA	1.5	1	1	2	1	1	9.3
Soler-Silva et al. (2021)	1	1.5	1.5	1	1	1	1	1	9.0
Stefanidis et al. (2012)	1	0.5	0.5	1	2	2	1	1.5	9.5
Stefanidis et al. (2013)	1	0.5	0.5	1	2	2	1	1	9.0
Supe et al. (2005)	1	NA	1.5	1	1	1	1	1	8.2
Sutton et al. (2017)	1	NA	0.5	3	2	2	1	1	11.5
Tamate et al. (2022)	2	NA	1	3	2	2	1	1.5	13.6
Tjalma et al. (2013)	1	NA	0.5	1	1	1	1	1	8.7
Udomsawaengsup et al. (2005)	1	0.5	0.5	1	1	2	1	1	8.0
Usami et al. (2018)	1	1	1.5	1	1	2	1	1	9.5
Vlaovic et al. (2008)	1.5	1.5	1.5	3	2	2	1	1.5	14.0
White et al. (2014)	1	NA	0.5	1	1	2	1	1	8.2
Wyles et al.	2	NA	1.5	3	2	2	1	1.5	14.2
Yiasemidou et al. (2017)	1	NA	0.5	1	1	2	1	1	8.2
Yoshida et al. (2021)	1	NA	0.5	1	2	2	1	1	9.3

The studies included in this review are summarized in Table 3, which outlines key details such as the specialty, laparoscopic procedure performed, type of body donor utilized, methodology, and outcome measures. Among the eligible studies, a significant disparity was present between trial design, complexity of skill being assessed, surgical specialty and outcome measures. As a result, we have separated the studies into 5 main groups for our discussion: (1) descriptive and feasibility studies assessing feasibility and face validity; (2) studies assessing trainee satisfaction and face validity; (3) studies assessing content validity; (4) studies assessing construct validity; and (5) comparative studies.

**Table 3:** Studies included in our review following the literature search

Author and Year	Place	Specialty	Laparoscopic procedures undertaken	Human body donor model used	Methodology	Outcome measure	Comparisons made	Participants	Model aspects assessed
Ackermann (2020a)	Germany	Obstetrics and gynaecology	Hysterectomy, adnexectomy, sacrocolpopexy, pectopexy, colposuspension, pelvic and para-aortic lymphadenectomy	Ethanol-glycerin-lysoformin embalmed	Questionnaire	Development of knowledge, development of surgical skills, perceived educational value, perceived satisfaction with model	n/a	73 participants	Content validity
Ackermann (2020b)	Germany	Obstetrics and gynaecology	Anatomical dissection	Ethanol-glycerin-lysoformin embalmed	Questionnaire	Perceived authenticity, perceived development of surgical skills, perceived educational value	Surgery on the real patient	208 participants (resident = 11; specialist = 24; consultant = 81; senior consultant = 44; clinical director = 40)	Content validity
AlJamal (2018)	USA	General Surgery	Endoscopic totally extraperitoneal inguinal hernia repair	Unspecified	Pre- and post-course tests, questionnaire	Development of knowledge, perceived educational value, perceived satisfaction with model	Constructed models	14 general surgery trainees	Content validity, face validity
Bilge and Celik (2017)	Türkiye	Unspecified	Basic laparoscopy practice, laparoscopic suturing and ligation, laparoscopic total hysterectomy, liver mobilisation, colectomy, jejunosigmoidostomy	Modified Larssen® solution	Description of experience, questionnaire	Perceived authenticity, perceived satisfaction with model	Fresh frozen, and F10 fixed	282 (252 trainees and 30 trainers)	Face validity
Blaschko et al. (2007)	USA	Cardiothoracic Surgery; Urology	Robot-assisted laparoscopic prostatectomy, cardiac surgery	Fresh frozen	Questionnaire	Perceived satisfaction with model	n/a	12 postgraduate urologists and 1 surgical assistant; 8 cardiothoracic surgeons and 1 surgical assistant	n/a
Britt et al. (2015)	USA	General Surgery	Laparoscopic suturing (on bowel)	Fresh	Post-simulation task performance	Change in operative time, development of skills	FLS simulated bowel	14 surgical trainees	n/a
Cundiff et al. (2001)	USA	Obstetrics and gynaecology	Pelvic dissection	Fresh	Post-simulation task performance, test scores, and questionnaire	Development of knowledge development of surgical skills, perceived educational value, perceived satisfaction with model	n/a	27 Gynaecology trainees (intervention (n) = 15)	Content validity, face validity
Danion et al. (2020)	France	General Surgery	Laparoscopic sleeve gastrectomy and laparoscopic roux-in-y bypass	SimLife®	Questionnaire	Perceived authenticity, perceived educational value, perceived satisfaction with model	n/a	24 surgical residents	Face validity
Donatini et al. (2021)	France	General Surgery	Laparoscopic adrenalectomy	SimLife®	Post-simulation task performance, questionnaire	Development of anatomical knowledge, development of surgical skills, perceived authenticity, perceived educational value, perceived satisfaction with model	n/a	n/a	Content validity, validity
Foster et al. (2014)	United Kingdom	General Surgery	ELAPE (Dissection of vascular pedicle, identification of ureter and gonadals, dissection of mesorectal plane, extralevator dissection etc.)	Fresh frozen	Objective specimen evaluation, questionnaire	Development of surgical skills, perceived authenticity, perceived development of competence, perceived educational value, perceived satisfaction with model	n/a	64 consultant colorectal surgeons	Content validity, face validity
Gamboa et al. (2009)	USA	Urology	Robot assisted laparoscopic prostatectomy	Unspecified	Questionnaire	Impact on clinical practice	n/a	42 urologists	n/a
Giger et al. (2008)	Switzerland	General Surgery	Left/right hemicolectomy, bilio-pancreatic diversion with duodenal switch or standard gastric bypass, hernia surgery: totally extraperitoneal approach (TEP), transabdominal preperitoneal approach (TAPP), laparoscopic infrarenal aorto-bifemoral bypass	Thiel embalmed	Questionnaire	Perceived authenticity, perceived educational value, perceived satisfaction with model	n/a	33 participants (31 consultant surgeons and 2 senior residents)	Face validity
He et al. (2014)	Australia	General Surgery	Laparoscopic Kidney Transplant	Fresh frozen	Description of experience	Feasibility of performing laparoscopic procedure	n/a	n/a	Content validity
Katz et al. (2003)	France	Urology	Extraperitoneal and retroperitoneal nephrectomy, transperitoneal nephrectomy	Fresh	Questionnaire	Perceived authenticity, perceived development of skills, perceived satisfaction with model	Anaesthetised pigs	7 urology trainees in the body donor model and 9 in the porcine	Face validity
Kong et al. (2023a)	United Kingdom	General Surgery	Dissection of retropubic space and pelvic side walls, intracorporeal and extracorporeal suturing of structures, monopolar diathermy, Bipolar diathermy and bipolar coagulation	Genelyn® embalmed	Description of experience	Perceived authenticity, perceived satisfaction with model	n/a	2 participants (1 expert and 1 novice laparoscopic surgeon)	Face validity
Leblanc et al. (2010a)	USA	General Surgery	Laparoscopic Sigmoid Colectomy	Fresh	Post simulation task performance, questionnaire	Development of surgical skills, perceived satisfaction with model	Virtual reality simulator (ProMIS®)	34 practicing surgeons (7 on human body donor; 27 on simulator)	Face validity
Leblanc et al. (2010b)	USA	General Surgery	Hand assisted laparoscopic sigmoid colectomy	Fresh	Post-simulation task performance questionnaire	Development of surgical skills, perceived satisfaction with model	Virtual reality simulator (ProMIS®)	34 practicing surgeons (7 on human body donor; 27 on simulator)	Face validity
Levine et al. (2006)	USA	Obstetrics and gynaecology	Bead transfer, and suturing a stuffed vinyl glove	Lightly embalmed	Post-simulation task performance	Change in operative time, development of surgical skills	n/a	29 Obstetrics and gynaecology residents	Content validity
Lewis et al. (2012)	USA	General Surgery	Liver harvest, pancreatectomy, abdominal aortic aneurysmectomy, groin herniorrhaphy, thyroidectomy, gastrectomy, thoracic outlet procedures, wound repair, intestinal anastomoses, abdominal incisions, and chest tubes, trauma exposures, neck exposures, and percutaneous tracheostomy	Fresh frozen	Questionnaire	Perceived development of surgical skills, perceived educational value, perceived satisfaction with model	n/a	40 surgical residents	n/a
Lim et al. (2018)	United Kingdom	Obstetrics and gynaecology	Tubal clip insertion, laparoscopic salpingectomy, Laparoscopic oophorectomy, Laparoscopic specimen retrieval and optimising surgical field (open and laparoscopic)	Fresh frozen	Questionnaire	Perceived development of surgical skills	n/a	9 Obstetrics and gynaecology trainees	Construct validity
Mantica et al. (2020)	Italy	Urology	Laparoscopic radical prostatectomy, laparoscopic partial nephrectomy, laparoscopic radical nephrectomy	Thiel embalmed	Questionnaire	Perceived authenticity, perceived development of surgical skills, perceived educational value, perceived satisfaction with model	n/a	40 urologists	Face validity
Marcu et al. (2021)	USA	Obstetrics and gynaecology	Laparoscopic dissection	Thiel embalmed	Pre- and post-course test scores, questionnaire	Development of anatomical knowledge, perceived development of surgical skills	n/a	44 participants (25 completed fellowship, 15 completed residency, 2 were residents, 2 were fellows)	Content validity
Miller et al. (2019)	USA	General Surgery	Laparoscopic adrenalectomy, laparoscopic appendectomy, laparoscopic cholecystectomy, laparoscopic hand-assisted right colectomy, laparoscopic inguinal hernia repair with mesh, laparoscopic Nissen fundoplication, laparoscopic splenectomy, laparoscopic ventral hernia repair with mesh, laparoscopic and open common bile duct exploration, open gastrectomy, open Hartmann’s sigmoidectomy with end colostomy, open pancreaticoduodenectomy, retrograde endovascular balloon occlusion of the aorta	Fresh frozen	Questionnaire	Perceived development of surgical skills	n/a	15 surgical residents and 5 surgeons	Construct validity, content validity
Morizane et al. (2022)	Japan	General Surgery; Obstetrics and gynaecology; Urology	Laparoscopic pelvic lymph node dissection	Thiel® embalmed	Description of experience	Feasibility of performing laparoscopic procedure	n/a	3 surgeons (1 from each specialty)	Face validity
Nagase et al. (2020)	Japan	Unspecified	Pneumoperitoneum and laparoscopic dissection	N-vinyl-2-pyrolidone embalmed	Description of experience	Feasibility of performing laparoscopic procedure	Formalin-embalmed	n/a	Face validity
Nagase et al. (2022)	Japan	Unspecified	Pneumoperitoneum, manipulation of the abdominal viscera	N-vinyl-2-pyrolidone embalmed	Description of experience	Feasibility of performing laparoscopic procedure	n/a	n/a	Face validity
Nebot-Cegarra and Macarulla-Sanz (2004)	Spain	Unspecified	Pneumoperitoneum	Unspecified embalmed	Description of experience	Feasibility of performing laparoscopic procedure	n/a	n/a	Face validity
Palter and Grantcharov (2012)	Canada	General Surgery	Laparoscopic right colectomy or laparoscopic sigmoid colectomy	Unspecified	Post-simulation task assessment, multiple choice test scores	Development of anatomical knowledge, development of surgical skills	n/a	24 surgical residents (18 post-intervention)	Content validity, predictive validity
Pattana-arun et al. (2005)	Thailand	General Surgery	Proctocolectomy including right colon, left colon, sigmoid colon and low anterior resection	Soft	Questionnaire	Perceived educational value, perceived satisfaction with model	n/a	14 experienced surgeons (8 colorectal surgeons)	n/a
Rai et al. (2015)	United Kingdom	Urology	Transperitoneal laparoscopic nephrectomy	Thiel® embalmed	Questionnaire	Perceived authenticity, perceived satisfaction with model	n/a	4 expert and 20 non-expert surgeons	Construct validity, content validity, face validity
Rai et al. (2012)	United Kingdom	Urology	Laparoscopic radical nephrectomy	Thiel® embalmed	Questionnaire	Perceived authenticity, perceived satisfaction with model	n/a	4 senior urology trainees and 4 fully trained urologists (faculty)	Construct validity, content validity, face validity
Rashidian et al. (2019)	Belgium	General Surgery	Laparoscopic hepatectomy	Thiel® embalmed	Questionnaire	Impact on clinical practice, perceived educational value	n/a	64 consultant surgeons	n/a
Ross (2008)	USA	General Surgery	Laparoscopic segmental colectomy	Unspecified	Questionnaire	Impact on clinical practice, perceived educational value	n/a	43 general surgeons	n/a
Ruiz-Tovar et al. (2019)	Spain	General Surgery	Roux-en-Y gastric bypass, sleeve gastrectomy	Thiel® embalmed	Questionnaire	Perceived authenticity, perceived educational value, perceived satisfaction with model	n/a	16 professors of bariatric surgery and 48 students	Face validity
Sanchez-Ferrer et al. (2019)	Spain	Obstetrics and gynaecology	Anterior compartments prolapse repair	Thiel® embalmed	Questionnaire	Perceived development of knowledge, perceived educational value	n/a	72 participants	Content validity
Sanchez-Ferrer et al. (2020)	Spain	Obstetrics and gynaecology	Neovagina surgery (modified McIndoe, modified Vecchietti, Davydov, and vulvoperineal pediculated flaps with simultaneous laparoscopic approach	Thiel® embalmed	Questionnaire	Perceived educational value	n/a	133 participants (majority gynaecologists)	n/a
Sharma and Horgan (2012)	United Kingdom	General Surgery	Laparoscopic sigmoid colectomy, laparoscopic incisional hernia repair, and basic laparoscopic tasks	Fresh frozen	Questionnaire	Perceived educational value, perceived satisfaction with model	Virtual reality stimulators (Lap Mentor)	45 participants	Construct validity
Sharma et al. (2013)	United Kingdom	General Surgery	Dominant to nondominant hand sharp peg transfer, nondominant to dominant hand sharp peg transfer, simulated appendicectomy, Intracorporeal knot tying, extracorporeal knot tying	Fresh frozen	Post-simulation task assessment	Development of surgical skills	n/a	19 junior surgical trainees (control = 9; body donor simulation = 10)	Content validity
Sharma et al. (2012)	United Kingdom	General Surgery	Dominant to nondominant hand sharp peg transfer, nondominant to dominant hand sharp peg transfer, simulated appendicectomy, Intracorporeal knot tying, extracorporeal knot tying	Fresh frozen	Post-simulation task assessment	Development of surgical skills	n/a	10 junior surgical trainees and 2 experts	Construct validity, predictive validity
Slieker et al. (2012)	Netherlands	General Surgery	Mobilisation of the small and large bowels, and exposure of central vessels and ureters	Anubifix® embalmed	Questionnaire	Perceived authenticity, perceived development of surgical skills, perceived educational value, perceived satisfaction with model	Animal models and virtual reality simulators	11 surgical residents	Face validity
Soler-Silva (2021)	Spain	General Surgery; Obstetrics and gynaecology; Urology	Urogynaecology surgery	Thiel® embalmed	Questionnaire	Perceived authenticity, perceived development of surgical skills, perceived educational value, perceived satisfaction with model	n/a	75 postgraduate surgeons	Face validity
Stefanidis et al. (2012)	USA	General Surgery	Upper GI and colorectal surgery	Unspecified	Questionnaire	Perceived educational value	Porcine model	16 general surgery residents, 7 general surgery faculty members	n/a
Stefanidis et al. (2013)	USA	General Surgery	Laparoscopic colectomy, laparoscopic cholecystectomy, laparoscopic ventral hernia repair, laparoscopic nephrectomy, laparoscopic Nissen fundoplication, laparoscopic Heller myotomy	Unspecified	Questionnaire	Perceived authenticity, perceived educational value, perceived satisfaction with model	Porcine model	39 participants (9 faculty and 30 residents)	Face validity
Supe et al. (2005)	India	General Surgery	Laparoscopic cholecystectomy, appendicectomy, splenectomy, intestinal explorations, varicocele-vein occlusion, and mesenteric lymph node biopsy	Unembalmed	Questionnaire	Perceived educational value, perceived satisfaction with model	n/a	32 participants (practicing surgeons)	n/a
Sutton et al. (2017)	USA	General Surgery	Laparoscopic nephrectomy	Lightly embalmed	Post-simulation task assessment	Change in operative time	n/a	n/a	Content validity
Tamate et al. (2022)	Japan	Obstetrics and gynaecology	Laparoscopic radical hysterectomy and laparoscopic pelvic exenteration	Thiel embalmed	Multiple choice test scores, questionnaire	Development of anatomical knowledge, perceived satisfaction with model	n/a	45 participants	Content validity
Tjalma et al. (2013)	Belgium	Obstetrics and gynaecology	Laparoscopic hysterectomy and pelvic lymphadenectomy	Thiel® embalmed	Questionnaire	Perceived satisfaction with model	n/a	27 gynaecologists	n/a
Udomsawaengsup et al. (2005)	Thailand	General Surgery	Laparoscopic upper gastrointestinal, colonic, and hepatobiliary surgery, splenectomy, and hernia repair, laparoscopic posterior truncal vagotomy with anterior highly selective vagotomy, laparoscopic cardiomyotomy, laparoscopic Nissen fundoplication, laparoscopic gastrojejunostomy, laparoscopic appendectomy, laparoscopic n/a Face colon mobilization, laparoscopic anterior resection laparoscopic cholecystectomy, laparoscopic liver dissection and laparoscopic distal pancreatectomy, Splenectomy, Hernia repair (totally extra peritoneal (TEP) and transabdominal preperitoneal repair (TAPP))	Soft	Questionnaire	Perceived satisfaction with model	n/a	31 participants (10 practicing surgeons and 21 residents) (+ 10 observers)	n/a
Usami et al. (2018)	Japan	Obstetrics and gynaecology	Laparoscopic radical hysterectomy, lymphadenectomy, bilateral salpingo-oophorectomy	Thiel® embalmed	Questionnaire	Perceived authenticity, perceived educational value, perceived satisfaction with model	n/a	17 expert laparoscopist obstetricians	Face validity
Vlaovic et al. (2008)	USA	Urology	Laparoscopic ablative renal surgery and laparoscopic reconstructive assisted prostatectomy	Unspecified	Post-simulation task assessment	Development of skills	n/a	101 urologists	Content validity
Wedel et al. (2019)	Germany	Obstetrics and gynaecology	Trocar placement and pneumoperitoneum, exploratory laparoscopy, anatomical plane dissection, electrosurgery suturing techniques	Ethanol-glycerin-lysoformin embalmed	Description of experience	Perceived authenticity of model, feasibility of performing laparoscopic procedure	n/a	n/a	Face validity
White et al. (2014)	United Kingdom	General Surgery	Laparoscopic liver resection	Fresh frozen	Questionnaire	Perceived educational value	Anaesthetised pigs	32 participants	n/a
Wyles (2011)	United Kingdom	General Surgery	Colorectal surgery	Fresh frozen	Questionnaire	Perceived authenticity, perceived educational value, perceived satisfaction with model	Anaesthetised pigs	103 surgeons (66 from body donor model and 37 from porcine model)	Construct validity, face validity
Yiasemidou et al. (2017)	United Kingdom	Unspecified	Any surgical procedure the surgeons wished to perform	Thiel® embalmed	Questionnaire	Perceived authenticity, perceived educational value, perceived satisfaction with model	n/a	27 participants (26 surgeons and 1 gastroenterologist)	Face validity
Yoshida et al. (2021)	Japan	Paediatric Surgery	Thoracoscopic esophagectomy, thoracoscopic right lower lobectomy, laparoscopic fundoplication, laparoscopic hepaticoduodenostomy and laparoscopic ureteroureterostomy	Saturated salt solution embalmed	Questionnaire	Perceived authenticity, perceived satisfaction with model	Anaesthetised pigs	9 participants, 3 teams consisting of a consultant paediatric surgeon, a senior trainee, and a junior trainee	Face validity

### Study characteristics

Our review included studies from 15 different countries. Over a quarter of the studies (*n* = 16) included in our review were form the USA. Over half of the studies were from Europe (*n* = 28). The USA and the UK together made up 49% of the studies included in our review.

51% of the studies focused on general surgery, with 22% centred on obstetrics and gynaecology and 11% on urology. The remaining studies included those without a specified specialty, multi-specialty training programs, and one study dedicated to paediatric surgery. These specialty distributions are summarized in Table 4.

**Table 4:** Study characteristics by specialty

Specialty	Total number of Studies (*n* = 55)
General Surgery	28
Obstetrics and gynaecology	12
Urology	6
Unspecified	5
Multi-specialty	3
Paediatric Surgery	1

15 different types of body donors were used in studies included in our review, as shown in Table 5. TEBDs and FFBDs were the most frequently utilized models, accounting for 27.3% and 20% of cases, respectively.

**Table 5:** Study characteristics by type of body donor used

Type of body donor	Number of studies (*n* = 55)
Thiel®	15
Fresh frozen	11
Unspecified	7
Fresh	5
Ethanol-Glycerin-Lysoformin	3
N-vinyl-2-pyrolidone	2
Lightly embalmed	2
SimLife®	2
Soft	2
Anubifix®	1
Genelyn®	1
Modified Larssen® Solution	1
Saturated salt solution embalmed	1
Unspecified embalmed	1
Unembalmed	1

### Descriptive studies assessing feasibility and face validity

A total of seven studies evaluated the feasibility of performing laparoscopic surgery and/or the face validity of donor models for laparoscopic simulation training by describing experiential insights with the models, without employing large cohort questionnaires or formal task assessments.

Bilge and Celik (2017) described the tissue characteristics of internal abdominal organs in modified Larssen® solution embalmed body donors as very similar to fresh tissues in FFBDs during laparoscopic interventions.

Kong et al. (2023a) reported the initial experience of an expert laparoscopic surgeon with Genelyn® embalmed models for laparoscopy training. This model was described to be suitable for establishment of pneumoperitoneum, and as having a surgical anatomy which was indistinguishable from in vivo models. There was minimal bad odour described in this model, as well as minimal need for suction. The consistency of tissue and ease of suturing as well as laparoscopic instrumentation were like the in vivo situation. The model was used for 3 weeks, and no differences were noticed between all the reported factors throughout the dissection sessions.

Morizane et al. (2022) demonstrated the face validity of TEBDs through assessment of lateral pelvic node dissections by surgeons belonging to three different specialties (general surgery, obstetrics and gynaecology, urology) which showed differences in procedures and the extent of node dissection in these three specialties.

Nebot-Cegarra and Sanz (2004) showed that as more abdominal muscular layers are sectioned in embalmed body donors, a greater increase in abdominal size is attained. Futhermore, this improves visualisation of the working space and eases surgical manipulation. This highlights novel a novel method to further optimise body donor models for use in laparoscopic surgical training.

Wedel et al. (2019) described their experience with ethanol-glycerol-lysoformin preserved body donors, stating that key laparoscopic tasks such as trocar handling, pneumoperitoneum, blunt/sharp dissection, removal of organs, electrosurgery, and suturing can be performed without difficulty. Wedel et al. (2019). They established that multiple reuses of the body donors over a year was feasible Wedel et al. (2019). They also found that their model necessitated higher levels of gas insufflation and increased energy for electrosurgery when compared to in vivo surgery Wedel et al. (2019).

### Studies assessing trainee satisfaction and face validity

The majority of studies fell within this category, with forty-two studies evaluating trainee satisfaction with various models. This was achieved by evaluation questionnaires that directly assessed satisfaction or measured perceived authenticity, perceived educational value, and perceived improvements in surgical skills or confidence scores.

Ackermann et al. (2020b)  showed that the assessment of participant satisfaction following Obstetrics and gynaecology training in ethanol-glycerol-lysoformin body donors yielded promising results, with authenticity being rated as 85.5% by participants. Participants, however, did not favour the replacement of real patients with donors; with only 14.5% agreeing that this would provide adequate training.

Britt et al. (2015) demonstrated that transitioning from the low fidelity box simulator to more realistic conditions using a fresh frozen body donor led to a > 333% increase in suturing times, showing the dramatic increase in mental workload associated with intracorporeal suturing in more realistic conditions.

Giger et al. (2008) assessed TEBDs for their use in laparoscopic bariatric, vascular, hernia, and colorectal surgery training revealed mean satisfaction scores of 4.0. for colorectal, 4.2 for hernia, 5.0 for bariatric, and 4.1 for vascular surgery. Participants also rated authenticity of tissue colour, consistency, and operative tactility highly (> 4.0) for all colorectal, hernia, and bariatric surgery, but these three parameters were rated only 2.8, 2.8, and 2.6, respectively, for the vascular course. The authors discussed the discrepancy in satisfaction rates between the other specialties and vascular surgery and suggested the need for perfused models for vascular surgery training. This is also an issue for other surgical specialties where realism of blood vessels is important, such as pelvic lymph node dissections, and the perfused SimLife® body donor model could be the way forward for training in these procedures, as reported by Danion et al. (2020); Donatini et al. (2021). This model, however, also has limitations as the revascularisation is achieved by a blood mimicking fluid and an extracorporeal pulse for practice device, meaning coagulation, platelet activation, and thrombin-derived products are not present as in the real-life situation. Danion et al. (2020); Donatini et al. (2021).

Pattana-arun et al. (2005) discussed the use of Thiel-embalmed body donors for laparoscopic proctocolectomy training, highlighting high levels of satisfaction among participants in Thailand. They noted two drawbacks: odour and air leakage through the ports, but the former was considered manageable, and the latter was easily addressed with purse-string sutures.

Rai et al. (2012) used TEBDs for laparoscopic radical nephrectomy training. The mean overall experience was rated 4.5 by trainees, and 4.3 by experienced laparoscopic surgeons on the five-point Likert scale.

Consultants involved in a laparoscopic liver surgery course, as reported by Rashidian et al. (2019) stated that training using these models was even more effective than proctoring in the operating room as a learning experience.

Ruiz-Tovar et al. (2019) assessed the perceived educational value of TEBDs for bariatric surgery in a separate study, showing that all 48 trainees participating in simulation training agreed that practicing on TEBDs should be mandatory before performing laparoscopic bariatric surgery in vivo; and all participants rated TEBD as a better model than animal models for laparoscopic bariatric surgery training. A greater elasticity of the bowel and stomach in the real patient was highlighted as the main drawback of the TEBD in this study.

Soler-Silva et al. (2021) showed that trainees found TEBDs beneficial for simulation training in pelvic floor and perineal surgical procedures, with 70% rating their use as “excellent” for improving anatomical knowledge, with all respondents agreeing that they allowed for the perfect recognition and dissection of tissues, with adequate elasticity enabling authentic manipulation of tissues.

Supe et al. (2005) at the use of unembalmed body donors for general surgery training found that although 96.9% of the participants rated the model as highly satisfactory. Trainees also suggested limitations to the use of these due to the absence of active bleeding, active breathing perception, and limited hours of working as the body donors tend to become malodourous after 6–8 h.

Udomsawaengsup et al. (2005) assessed the perceptions of surgical staff and residents, rating anatomical quality of tissue as an average of 4.72, and their use for laparoscopic surgical procedures as an average of 4.80. Some domains that scored poorly were unflavoured smelling and air leakage.

Usami et al. (2018) investigated the use of TEBDs for Obstetrics and gynaecology training and showed that although surgeons were very satisfied with the authenticity of the uterus, adnexa, and the ureter in this model, large blood vessels were rated as less lifelike, most likely due to the lack of perfusion in this model.

### Studies assessing content validity

A total of eighteen studies evaluated the content validity of various body donor models, utilizing methods such as post-simulation task assessments, post-simulation knowledge testing, questionnaires, or descriptive analyses of experiences with individual models.

Levine et al. (2006) demonstrated that body donor models effectively enhanced laparoscopic technical skills among obstetrics and gynaecology trainees, as measured by their performance on a set of tasks using a laparoscopic simulator.

Marcu et al. (2021) used TEBDs to evaluate their potential for the development of knowledge in laparoscopic surgery for obstetrics and gynaecology. A multiple-choice test was undertaken following a multiday laparoscopic body donor pelvic neuroanatomy course. The post-course anatomical knowledge test results were significantly higher than the pre-course test results.

The improvement in surgical skills in performing laparoscopic donor nephrectomies after training with lightly embalmed body donors was analysed by Sutton et al. (2017) showed that with training using lightly embalmed body donors, time taken for dissection of gonadal vein, ureter, renal hilum, adrenal and lumbrical veins, simulated warm ischemic time (WIT), and overall operative time all decreased.

Vlaovic et al. (2008) used a training programme consisting of didactic lectures, pelvic trainer and VR simulator practice, and animal and body donor simulation sessions improved laparoscopic skills of ring transfer, suture threading, cutting, suturing, and overall score.

### Studies assessing construct validity

A total of eight studies assessed construct validity of various body donor models.

Miller et al. (2019) showed that using human body donors increased confidence scores in  general surgery residents at all levels, but this was least significant in first year residents, possibly suggestive of an increase in cognitive load because of a more dramatic transition to a high-fidelity model. This was still statistically significant however, and therefore still suggesting great benefit of training in these models to first year residents.

Rai et al. (2015) showed that TEBDs have construct validity when used for training in laparoscopic radical nephrectomy through comparing video recordings of the procedure being performed by 4 expert surgeons and 20 non-expert surgeons.

Sharma et al. (2012) found that FFBDs have construct validity through comparing the performances of novice and expert surgeons for several tasks including such as intracorporeal and extracorporeal knot tying and simulated appendectomy.

### Comparative studies

Fifteen studies were comparative, assessing the body donor model alongside alternative models such as other body donors, live anesthetized pigs, porcine models, or virtual reality simulations.

Cundiff et al. (2001) found no significant differences in faculty evaluations or test scores assessing surgical anatomy knowledge between a group performing laparoscopic pelvic dissections in fresh body donors and a control group. However, the intervention group reported higher satisfaction rates with the anatomy training.

Katz et al. (2003) compared how delegates perceived FFBDs versus live anesthetized pigs on laparoscopic urological procedures showed similar satisfaction with both methods but found that the fresh body donors provided a greater understanding of anatomy, instrumentation, and laparoscopic techniques. Trainees rated their general satisfaction with the body donor model as 4.14 on the five-point Likert scale. Notably, the rating given to FFCs by this cohort was less than the rating given to TEBDs by a different cohort reported by Rai et al (2012).

 Wyles et al. (2011) compared trainee/trainer satisfaction for colorectal surgery with FFBDs and live anaesthetised porcine models. The participants rated the FFBD model significantly superior for realistic simulation for port placement and anatomy on the five-point Likert scale. Trainees, however, rated the porcine model significantly better in terms of tissue quality, persistence of air leak, disturbance by odour. Delegates thought perfusion in live porcine models did not contribute to quality of training and thought both models were superior to virtual reality training.

Nagase et al. (2020) compared two different types of body donors for surgical training in the same cohort. As per the study, pyrrolidone embalmed body donors had softer abdomens compared to the formalin-embalmed body donors, making it easier to move the laparoscope in the peritoneal space. Due to its softer anatomy, the changes in abdominal diameter and circumference in response to CO_2_ insufflation was significantly greater in the pyrrolidone group, which possibly suggests its better suitability than formalin embalmed body donors for laparoscopic simulation training.

Leblanc et al. (2010a; b) assessed trainees using virtual reality and fresh body donor models in laparoscopic straight and hand-assisted colonic surgery through objective structured assessment forms that evaluated technical skills, events, and satisfaction. They concluded from an analysis of the scores found suggested that delegates found body donor models more difficult but had more satisfaction with them Leblanc et al. (2010a; b).

Slieker et al. (2012) introduced the Anubifix® embalming method, a novel light embalming technique using a 4% formalin solution designed to prevent false vessel coloration during fixation. They reported high satisfaction with this model due to its realistic color, anatomical accuracy, tissue consistency, and superiority over animal and virtual reality models.

Sharma et al. 2013 found that participants who were assigned to practice technical tasks on fresh frozen body donors demonstrated better cautery safety and a reduced path length of instruments on a virtual reality simulator compared to those in the control group. Additionally, the group that practiced on fresh frozen body donors also showed a reduced number of bowel perforations, which was higher than the control group prior to simulation training.

Yoshida et al. (2021) looked at the use of saturated salt solution embalmed body donors for training in paediatric surgery, comparing these to anaesthetised pig models. After evaluation by 3 teams, each consisting of 3 surgeons, the body donor models were found to score significantly higher than live pig models for anatomical relationships, operative field, and organ size: in the context of laparoscopic fundoplication training. However, across a range of 5 different surgical procedures, they reported the median score for tissue texture to be significantly lower than for other criteria.

Only one study assessed predictive validity of models through transition to the real life patient. Palter and Grantcharov (2012). The study demonstrated that a specific training curriculum, which included VR simulator training, cognitive training, and body donor lab sessions, resulted in significantly better performance on the VR simulator compared to the control group. The control group underwent the conventional training pathway typically followed in standard residency programs. Notably, these improvements extended beyond the simulator: trainees in the specific curriculum also performed significantly better in laparoscopic right hemicolectomy procedures on real patients, as assessed by senior surgeons during live operations that were blinded as to the randomisation of the residents.

## Discussion

In this review, we examined various body donor preparation techniques, including fresh frozen and tissue-embalmed models, assessing their strengths, limitations, and applications across surgical specialties. The discussion explores these methods in detail, with a focus on their tissue authenticity and relevance to specific training needs. Furthermore, we address potential innovations and highlight gaps in the literature that warrant further investigation.

FFBDs and soft embalmed cadavers are widely utilized in surgical simulation training and are often regarded by trainees as high-fidelity models for replicating human anatomy and surgical procedures. However, the current evidence base lacks robust comparative analyses between these models, particularly when assessing dimensions beyond face validity, such as construct validity, biomechanical fidelity, and educational outcomes. Our literature search found only two studies comparingdifferent types of body donor models. Bilge and Celik (2017); Nagase et al. (2020). Both are descriptive studies lacking quantitative and objective data; however, they suggest that the physical properties of soft embalmed cadavers are comparable to FFBDs. Further research and comparative studies may be beneficial to fully understand the nuanced differences between FFBDs and soft embalmed body donors in surgical training. Such studies could help identify the most appropriate body donor model for different training objectives and specialties, ultimately enhancing the effectiveness of surgical simulation programs.

FFBDs are recognised for their high authenticity and usefulness in training, as indicated by their consistently high ratings for educational value. Participants in studies using FFBDs were perceived highly for authenticity, particularly in terms of replicating surgical procedures accurately. However, FFBDs occasionally received criticism for unpleasant odour and persistent air leak, which could distract from the overall training experience Vlaovic et al (2008); Wedel et al.  (2019).

On the other hand, soft embalmed body donors also received positive feedback, especially regarding their suitability for specific surgical procedures. Participants rate TEBDs highly for authenticity in terms of tissue characteristics relevant to various surgeries. Despite this, TEBDs were also noted to have drawbacks, especially regarding their lack of realistic vascular tissue in several studies Usami et al 2018. Yiasemidou et al 2017. The lack of perfusion in TEBDs can cause negative effects in other aspects as well, for example due to the appearance of a paler mucosa than in vivo. The Simlife® and Anubifix® embalmed donor models can be used to get around this problem where the authenticity of the vasculature is key to the quality of training. Danion et al. (2020); Donatini et al. (2021); Slieker et al. (2012).

Other soft embalmed models are also promising for use in laparoscopic training owing to their unique characteristics. Formalin-embalmed body donors have been widely utilised in surgical training due to their availability and effective preservation of tissue structure. However, their inherent stiffness and altered tissue properties are often seen as limitations, particularly for laparoscopic simulations. Interestingly, this characteristic could be advantageous for certain procedures, such as pelvic floor surgery, where the preservation of muscle tonus is crucial for maintaining realism. Many alternative embalming methods, which are associated with excessive reduction in muscle tone, may inadvertently lower the fidelity in these procedures. This remains a challenging aspect because high muscle tone, in contrast, may hinder establishment or maintenance of pneumoperitoneum in the specific context of laparoscopic surgery. Nagase et al. (2020).

Use of embalmed body donors may also be of great benefit for training in laparoscopic surgery in the paediatric setting. Yoshida et al. (2021). While animal models, such as rabbits and pigs, are commonly used for training in minimally invasive surgery (MIS), they often fail to accurately replicate the size and anatomical characteristics of paediatric patients. Yoshida et al. (2021). Rabbits, despite their size similarity to neonates, present significant limitations due to differences in tissue properties, such as connective tissue and susceptibility to aspiration pneumonia, specifically impacting their utility for oesophageal procedures. Yoshida et al. (2021). Similarly, while pigs offer better respiratory function, their anatomical differences, including a large multilobulated liver and elongated intestines, complicate their use for paediatric surgery training. Yoshida et al. (2021). In contrast, embalmed body donors provide a more realistic alternative, although at a higher cost and with some preservation challenges. Yoshida et al. (2021). Moreover, the limited availability of body donors that are size and age congruent to the paediatric population underscores the importance of utilizing alternative models.

Although evidence surrounding body donor models suggests that they offer significant advantages for postgraduate surgical trainees, particularly in terms of anatomical realism and the opportunity to practice complex procedures, the role of low-fidelity models should not be underestimated. While they may lack the anatomical accuracy of body donor models, they provide a cost-effective and accessible alternative, particularly for mastering basic skills and techniques. Massoth et al. (2019); Reedy (2015). The integration of both high-fidelity body donor models and low-fidelity simulations can offer a comprehensive training experience, ensuring that trainees develop both foundational and advanced surgical skills. Cognitive load theory emphasizes the benefits of low-fidelity models in reducing task complexity (intrinsic load) and external distractions (extraneous load), particularly for early-stage learners such as medical students and novice surgical trainees. Reedy (2015); Kong et al. (2023b). This is supported by AlJamal et al. (2018), which demonstrated that general surgery trainees preferred low-cost models for initial learning and understanding of endoscopic totally extraperitoneal inguinal hernia repair. However, body donor models were favoured in terms of the overall training experience. AlJamal et al. (2018). Similarly, Wyles et al. (2011) found that surgical trainees rated porcine models  highly for familiarization with laparoscopic instruments, although they identified the FFBD as the superior overall training model.

While our review primarily focuses on the educational outcomes of various body donor models, issues surrounding associated costs and logistical challenges are important considerations as well. Embalmed body donor models, even with soft embalming techniques, have been shown to remain suitable for prolonged use, with no perceived deterioration in tissue quality after 3 weeks of sequential use which are not possible with fresh frozen models. Kong et al. (2023a. Although body donor models are generally more expensive than lower-fidelity alternatives, these costs can be mitigated through planned and coordinated use across multiple specialties. Blaschko et al. (2007).

The cost of N-vinyl-2-pyrrolidone embalming is comparable to that of Thiel® embalming, at approximately $260. Nagase et al. (2022); Hayashi et al. (2016). Evidence also suggests a cost advantage for Genelyn® embalming over Thiel® embalming, at $200. Rajasekhar et al. (2021). Interestingly, Rajasekhar et al. reported the cost of Thiel® embalming to be $1000, significantly more expensive than the amount reported by Hayashi et al. Hayashi et al. (2016); Rajasekhar et al. (2021). In contrast, formalin embalming is significantly less expensive, costing approximately $13 per cadaver, while saturated saline solution embalming costs $26. Hayashi et al. (2016). Larssen embalming costs $70 for chemicals, with an additional $55 required for annual storage. Bilge and Celik (2017). While such costs analyses exist and suggest the higher costs of soft embalmed techniques versus fresh frozen models, these are often not integrated within formal cost–benefit analyses especially if sequential prolonged used is employed within soft embalmed models. Broader related perspectives such as ecological impact and sustainability are issues of increasing importance in the global context in need of further good quality data.

Limitations of this review include the possibility of outcome reporting bias, as all the feasibility studies that came up in our literature search which were relevant to our review reported procedures that proved to be feasible in body donor models. Furthermore, there were not many disadvantages reported about any of the body donor models. Moreover, as previously discussed, the comparative analysis between different body donor models in our review relied predominantly on qualitative studies, with limited objective evaluation to provide more robust evidence. Furthermore, only one study included in our review examined the predictive validity of skills demonstrated in the body donor model translating to performance in the operating theatre setting Palter and Grantcharov (2012). This highlights a gap in the current literature, underscoring the need for more quantitative research to better assess the long-term effectiveness and transferability of training outcomes from body donor models to clinical practice.

Moreover, one of the challenges in comparing different body donor models used in laparoscopic training is the inconsistency in outcome measures across studies. The majority of studies assess trainee satisfaction using questionnaires. Only a minority of studies assessed content validity and predictive validity. Even within the latter group of studies, there is heterogeneity in how this is assessed including measures such as change in operating times and objective examiner evaluations. This heterogeneity makes synthesis and inter-study comparisons challenging. There is an important need for standardised evaluation and outcome reporting of simulation models.

Ultimately, body donor models are valuable tools for surgical simulation, but further research is needed to evaluate other important non-educational outcomes, such as sustainability and cost-effectiveness, as well as to provide robust comparative evidence on different body donor models.

## Supplementary Information

Below is the link to the electronic supplementary material.

Supplementary file1 (DOCX 15 KB)

## Data Availability

A data availability statement is not applicable.

## References

[CR1] Ackermann J, Wedel T, Hagedorn H et al (2020a) Establishment and evaluation of a training course in advanced laparoscopic surgery based on human body donors embalmed by ethanol-glycerol-lysoformin fixation. Surg Endosc 35(3):1385–1394. 10.1007/s00464-020-07523-6 32444969 10.1007/s00464-020-07523-6 PMC7886762

[CR2] Ackermann J, Wedel T, Holthaus B et al (2020b) Didactic benefits of surgery on body donors during live surgery events in minimally invasive surgery. J Clin Med 9(9):2912. 10.3390/jcm9092912 32917056 10.3390/jcm9092912 PMC7563950

[CR4] Al Asmri M, Haque MS, Parle J (2023) A modified medical education research study quality instrument (MMERSQI) developed by Delphi consensus. BMC Med Educ 23(1):63. 10.1186/s12909-023-04033-6 36698117 10.1186/s12909-023-04033-6 PMC9878889

[CR5] AlJamal Y, Buckarma E, Ruparel R, Allen S, Farley D (2018) Cadaveric dissection vs homemade model: what is the best way to teach endoscopic totally extraperitoneal inguinal hernia repair? J Surg Educ 75(3):787–791. 10.1016/j.jsurg.2017.09.003 28970180 10.1016/j.jsurg.2017.09.003

[CR6] Bilge O, Celik S (2017) Cadaver embalming fluid for surgical training courses: modified Larssen solution. Surg Radiol Anat 39(11):1263–1272. 10.1007/s00276-017-1865-4 28497162 10.1007/s00276-017-1865-4

[CR7] Blaschko SD, Brooks HM, Dhuy SM, Charest-Shell C, Clayman RV, McDougall EM (2007) Coordinated multiple cadaver use for minimally invasive surgical training. JSLS 11(4):403–407 18237501 PMC3015855

[CR8] Britt RC, Scerbo MW, Montano M, Kennedy RA, Prytz E, Stefanidis D (2015) Intracorporeal suturing: transfer from fundamentals of laparoscopic surgery to cadavers results in substantial increase in mental workload. Surgery 158(5):1428–1433. 10.1016/j.surg.2015.03.032 26003907 10.1016/j.surg.2015.03.032

[CR9] Cundiff GW, Weidner AC, Visco AG (2001) Effectiveness of laparoscopic cadaveric dissection in enhancing resident comprehension of pelvic anatomy. J Am Coll Surg 192(4):492–497. 10.1016/s1072-7515(01)00815-8 11294406 10.1016/s1072-7515(01)00815-8

[CR10] Danion J, Donatini G, Breque C, Oriot D, Richer JP, Faure JP (2020) Bariatric surgical simulation: evaluation in a pilot study of SimLife, a new dynamic simulated body model. Obes Surg 30(11):4352–4358. 10.1007/s11695-020-04829-1 32621055 10.1007/s11695-020-04829-1 PMC7333933

[CR11] Donatini G, Bakkar S, Leclere FM et al (2021) SimLife model: introducing a new teaching device in endocrine surgery simulation. Updates Surg 73(1):289–295. 10.1007/s13304-020-00871-x 32876883 10.1007/s13304-020-00871-x PMC7464064

[CR12] Eisma R, Wilkinson T (2014) From, “silent teachers” to models. PLoS Biol 12(10):e1001971. 10.1371/journal.pbio.1001971 25333490 10.1371/journal.pbio.1001971 PMC4205111

[CR13] Foster JD, Gash KJ, Carter FJ et al (2014) Development and evaluation of a cadaveric training curriculum for low rectal cancer surgery in the English LOREC National Development Programme. Colorectal Dis 16(9):O308–O319. 10.1111/codi.12576 24460775 10.1111/codi.12576

[CR14] Gallagher AG, Ritter EM, Satava RM (2003) Fundamental principles of validation, and reliability: rigorous science for the assessment of surgical education and training. Surg Endosc 17(10):1525–1529. 10.1007/s00464-003-0035-4 14502403 10.1007/s00464-003-0035-4

[CR15] Gamboa AJ, Santos RT, Sargent ER et al (2009) Long-term impact of a robot assisted laparoscopic prostatectomy mini fellowship training program on postgraduate urological practice patterns. J Urol 181(2):778–782. 10.1016/j.juro.2008.10.018 19091351 10.1016/j.juro.2008.10.018

[CR16] Giger U, Frésard I, Häfliger A, Bergmann M, Krähenbühl L (2008) Laparoscopic training on Thiel human cadavers: a model to teach advanced laparoscopic procedures. Surg Endosc 22(4):901–906. 10.1007/s00464-007-9502-7 17704868 10.1007/s00464-007-9502-7

[CR17] Hayashi S, Naito M, Kawata S et al (2016) History and future of human cadaver preservation for surgical training: from formalin to saturated salt solution method. Anat Sci Int 91(1):1–7. 10.1007/s12565-015-0299-5 26670696 10.1007/s12565-015-0299-5

[CR18] He B, Mou L, Delriviere L, Hamdorf J (2014) A human cadaver model for laparoscopic kidney transplant. Exp Clin Transplant 12(1):21–24. 10.6002/ect.2013.0173 24471719 10.6002/ect.2013.0173

[CR19] Jaung R, Cook P, Blyth P (2011) A comparison of embalming fluids for use in surgical workshops. Clin Anat 24(2):155–161. 10.1002/ca.21118 21322038 10.1002/ca.21118

[CR20] Katz R, Hoznek A, Antiphon P, Van Velthoven R, Delmas V, Abbou CC (2003) Cadaveric versus porcine models in urological laparoscopic training. Urol Int 71(3):310–315. 10.1159/000072684 14512654 10.1159/000072684

[CR21] Kong CY, Fogg QA, Allam M (2023a) A novel model for hands-on laparoscopic pelvic surgery training on Genelyn-embalmed body: an initial feasibility study. Anat Sci Int 98(1):89–98. 10.1007/s12565-022-00677-4 35750974 10.1007/s12565-022-00677-4

[CR22] Kong CY, Iddles E, Glen P (2023b) A paper-based simulation model for teaching inguinal hernia anatomy. World J Surg 47(8):1842–1849. 10.1007/s00268-023-07018-0 37099135 10.1007/s00268-023-07018-0 PMC10132405

[CR23] Leblanc F, Champagne BJ, Augestad KM et al (2010a) A comparison of human cadaver and augmented reality simulator models for straight laparoscopic colorectal skills acquisition training. J Am Coll Surg 211(2):250–255. 10.1016/j.jamcollsurg.2010.04.002 20670864 10.1016/j.jamcollsurg.2010.04.002

[CR24] Leblanc F, Senagore AJ, Ellis CN et al (2010b) Hand-assisted laparoscopic sigmoid colectomy skills acquisition: augmented reality simulator versus human cadaver training models. J Surg Educ 67(4):200–204. 10.1016/j.jsurg.2010.06.004 20816353 10.1016/j.jsurg.2010.06.004

[CR25] Levine RL, Kives S, Cathey G et al (2006) The use of lightly embalmed (fresh tissue) cadavers for resident laparoscopic training. J Minim Invasive Gynecol 13(5):451–456. 10.1016/j.jmig.2006.06.011 16962531 10.1016/j.jmig.2006.06.011

[CR26] Lewis CE, Peacock WJ, Tillou A, Hines OJ, Hiatt JR (2012) A novel cadaver-based educational program in general surgery training. J Surg Educ 69(6):693–698. 10.1016/j.jsurg.2012.06.013 23111032 10.1016/j.jsurg.2012.06.013

[CR27] Lim CP, Roberts M, Chalhoub T, Waugh J, Delegate L (2018) Cadaveric surgery in core gynaecology training: a feasibility study. Gynecol Surg 15(1):4. 10.1186/s10397-017-1034-0. (**[Published correction appears in Gynecol Surg. 2018;15(1):5. 10.1186/s10397-018-1038-4]**) 29386989 10.1186/s10397-017-1034-0 PMC5770503

[CR28] Lloyd GM, Maxwell-Armstrong C, Acheson AG (2011) Fresh frozen cadavers: an under-utilized resource in laparoscopic colorectal training in the United Kingdom. Colorectal Dis 13(9):e303–e304. 10.1111/j.1463-1318.2011.02610.x 21689303 10.1111/j.1463-1318.2011.02610.x

[CR29] Mantica G, Pini G, De Marchi D et al (2020) Intensive simulation training on urological mini-invasive procedures using Thiel-embalmed cadavers: the IAMSurgery experience. Arch Ital Urol Androl. 10.4081/aiua.2020.2.93 32597107 10.4081/aiua.2020.2.93

[CR30] Marcu I, Balica A, Gavard JA et al (2021) Closing the knowledge gap in pelvic neuroanatomy: assessment of a cadaveric training program. BMC Med Educ 21(1):26. 10.1186/s12909-020-02443-4 33413351 10.1186/s12909-020-02443-4 PMC7792346

[CR31] Massoth C, Röder H, Ohlenburg H et al (2019) High-fidelity is not superior to low-fidelity simulation but leads to overconfidence in medical students. BMC Med Educ 19(1):29. 10.1186/s12909-019-1464-7 30665397 10.1186/s12909-019-1464-7 PMC6341720

[CR32] Miller A, Ali J, Veith JP, Uecker J, Ali S, Aydelotte J (2019) Combining instructional videos with cadaver training for general surgery residents. Am Surg 85(4):e230–e232 31043223

[CR33] Miskovic D, Wyles SM, Ni M, Darzi AW, Hanna GB. Systematic review on mentoring and simulation in laparoscopic colorectal surgery [published correction appears in Ann Surg. 2011 Feb;253(2):384]. *Ann Surg*. 2010;252(6):943–951. 10.1097/SLA.0b013e3181f662e5 10.1097/SLA.0b013e3181f662e5 21107103

[CR34] Morizane S, Honda M, Kihara K et al (2022) Laparoscopic pelvic lymph node dissection in cadaver surgical training from the combined perspectives of urologists, gastroenterologists and gynecologists improves overall knowledge and technique: initial experience of multidisciplinary cadaver surgical training at a single institution in Japan. Anat Sci Int 97(3):303–306. 10.1007/s12565-022-00655-w 35258811 10.1007/s12565-022-00655-w

[CR35] Nagase M, Kimoto Y, Sunami E, Matsumura G (2020) A new human cadaver model for laparoscopic training using N-vinyl-2-pyrrolidone: a feasibility study. Anat Sci Int 95(1):156–164. 10.1007/s12565-019-00494-2 31347090 10.1007/s12565-019-00494-2

[CR36] Nagase M, Nagase T, Tokumine J, Saito K, Sunami E, Shiokawa Y, Matsumura G (2022) Formalin-free soft embalming of human cadavers using N-vinyl-2-pyrrolidone: perspectives for cadaver surgical training and medical device development. Anat Sci Int 97(3):273–282. 10.1007/s12565-022-00664-9 35460067 10.1007/s12565-022-00664-9

[CR37] Nebot-Cegarra J, Macarulla-Sanz E (2004) Improving laparoscopy in embalmed cadavers: a new method with a lateral abdominal wall muscle section. Surg Endosc 18(7):1058–1062. 10.1007/s00464-003-9229-z 15156379 10.1007/s00464-003-9229-z

[CR38] Page MJ, McKenzie JE, Bossuyt PM et al. The PRISMA 2020 statement: an updated guideline for reporting systematic reviews. *BMJ*. 2021;372:n71. Published 2021 Mar 29. 10.1136/bmj.n71 10.1136/bmj.n71 PMC8005924 33782057

[CR39] Palter VN, Grantcharov TP (2012) Development and validation of a comprehensive curriculum to teach an advanced minimally invasive procedure: a randomized controlled trial. Ann Surg 256(1):25–32. 10.1097/SLA.0b013e318258f5aa 22664557 10.1097/SLA.0b013e318258f5aa

[CR40] Pattana-arun J, Udomsawaengsup S, Sahakitrungruang C, Tansatit T, Tantiphlachiva K, Rojanasakul A (2005) The new laparoscopic proctocolectomy training (in soft cadaver). J Med Assoc Thai 88(Suppl 4):S65–S69 16623005

[CR41] Pattanshetti VM, Pattanshetti SV (2010) Laparoscopic surgery on cadavers: a novel teaching tool for surgical residents. ANZ J Surg 80(10):676–678. 10.1111/j.1445-2197.2010.05454.x 21061747 10.1111/j.1445-2197.2010.05454.x

[CR42] Piechaud PT, Pansadoro A (2006) Transfer of skills from the experimental model to the patients. Curr Urol Rep 7(2):96–99. 10.1007/s11934-006-0066-1 16526992 10.1007/s11934-006-0066-1

[CR43] Rai BP, Tang B, Eisma R, Soames RW, Wen H, Nabi G (2012) A qualitative assessment of human cadavers embalmed by Thiel’s method used in laparoscopic training for renal resection. Anat Sci Educ 5(3):182–186. 10.1002/ase.1267 22362548 10.1002/ase.1267

[CR44] Rai BP, Stolzenburg JU, Healy S et al (2015) Preliminary validation of Thiel embalmed cadavers for laparoscopic radical nephrectomy. J Endourol 29(5):595–603. 10.1089/end.2014.0719 25565549 10.1089/end.2014.0719

[CR45] Rajasekhar SSSN, Kumar VD, Raveendranath V et al (2021) Advanced training in laparoscopic gastrointestinal surgical procedures using Genelyn®-embalmed human cadavers: a novel model. J Minim Access Surg 17(4):495–501. 10.4103/jmas.JMAS_152_20 33605926 10.4103/jmas.JMAS_152_20 PMC8486066

[CR46] Rashidian N, Willaert W, Giglio MC et al (2019) Liver surgery training course on Thiel-Embalmed human cadavers: program evaluation, trainer’s long-term feedback and steps forward. World J Surg 43(11):2902–2908. 10.1007/s00268-019-05103-x 31375870 10.1007/s00268-019-05103-x

[CR47] Reddy R, Iyer S, Pillay M, Thankappan K, Ramu J (2017) Soft embalming of cadavers for training purposes: optimising for long-term use in tropical weather. Indian J Plast Surg 50(1):29–34. 10.4103/ijps.IJPS_219_16 28615807 10.4103/ijps.IJPS_219_16 PMC5469231

[CR48] Reedy G (2015) Using cognitive load theory to inform simulation design and practice. Clin Simul Nurs 11:355–360. 10.1016/j.ecns.2015.05.004

[CR49] Ross HM, Simmang CL, Fleshman JW, Marcello PW (2008) Adoption of laparoscopic colectomy: results and implications of ASCRS hands-on course participation. Surg Innov 15(3):179–183. 10.1177/1553350608322100 18757376 10.1177/1553350608322100

[CR50] Rueda Esteban RJ, López-McCormick JS, Rodríguez-Bermeo AS, Andrade M, Hernández Restrepo JD, Targarona Soler EM (2023) Face, content, and construct validity evaluation of simulation models in general surgery laparoscopic training and education: a systematic review. Surg Innov 30(2):251–260. 10.1177/15533506221123704 36062557 10.1177/15533506221123704

[CR51] Ruiz-Tovar J, Prieto-Nieto I, García-Olmo D et al (2019) Training Courses in Laparoscopic Bariatric Surgery on Cadaver Thiel: Results of a Satisfaction Survey on Students and Professors. Obes Surg 29(11):3465–3470. 10.1007/s11695-019-04003-2. (**[Published correction appears in Obes Surg. 2019 Nov;29(11):3471. 10.1007/s11695-019-04050-9]**) 31168719 10.1007/s11695-019-04003-2

[CR52] Samia H, Khan S, Lawrence J, Delaney CP (2013) Simulation and its role in training. Clin Colon Rectal Surg 26(1):47–55. 10.1055/s-0033-1333661 24436648 10.1055/s-0033-1333661 PMC3699140

[CR53] Sanchez-Ferrer ML, Fernández-Andres Í, Martínez-Escoriza JC, Romero-Maroto J, Sánchez Del Campo F, Gómez-Pérez L (2019) Can the simultaneous laparoscopic approach improve the learning of vaginal surgery with meshes in the anatomical model? Neurourol Urodyn 38(7):1812–1817. 10.1002/nau.24082 31274225 10.1002/nau.24082

[CR54] Sanchez-Ferrer ML, Grimbizis G, Nisolle M et al (2020) Could training in an anatomical model be useful to teach different neovagina surgical techniques? A descriptive study about knowledge and experience of techniques for neovagina surgery. J Clin Med 9(11):3722. 10.3390/jcm9113722 33228242 10.3390/jcm9113722 PMC7699514

[CR55] Schiavo JH (2019) PROSPERO: an international register of systematic review protocols. Med Ref Serv Q 38(2):171–180. 10.1080/02763869.2019.1588072 31173570 10.1080/02763869.2019.1588072

[CR56] Sharma M, Horgan A (2012) Comparison of fresh-frozen cadaver and high-fidelity virtual reality simulator as methods of laparoscopic training. World J Surg 36(8):1732–1737. 10.1007/s00268-012-1564-6 22484566 10.1007/s00268-012-1564-6

[CR57] Sharma M, Macafee D, Pranesh N, Horgan AF (2012) Construct validity of fresh frozen human cadaver as a training model in minimal access surgery. JSLS 16(3):345–352. 10.4293/108680812X13462882735818 23318058 10.4293/108680812X13462882735818 PMC3535798

[CR58] Sharma M, Macafee D, Horgan AF (2013) Basic laparoscopic skills training using fresh frozen cadaver: a randomized controlled trial. Am J Surg 206(1):23–31. 10.1016/j.amjsurg.2012.10.037 23623462 10.1016/j.amjsurg.2012.10.037

[CR59] Slieker JC, Theeuwes HP, van Rooijen GL, Lange JF, Kleinrensink GJ (2012) Training in laparoscopic colorectal surgery: a new educational model using specially embalmed human anatomical specimen. Surg Endosc 26(8):2189–2194. 10.1007/s00464-012-2158-y 22286275 10.1007/s00464-012-2158-y PMC3392504

[CR60] Soler-Silva Á, Sanchís-López A, Sánchez-Guillén L et al (2021) The Thiel cadaveric model for pelvic floor surgery: Best rated in transferable simulation-based training for postgraduate studies. Eur J Obstet Gynecol Reprod Biol 256:165–171. 10.1016/j.ejogrb.2020.11.023 33248374 10.1016/j.ejogrb.2020.11.023

[CR61] Stefanidis D, Coker AP, Green JM, Casingal VP, Sindram D, Greene FL (2012) Feasibility and value of a procedural workshop for surgery residents based on phase II of the APDS/ACS national skills curriculum. J Surg Educ 69(6):735–739. 10.1016/j.jsurg.2012.06.009 23111039 10.1016/j.jsurg.2012.06.009

[CR62] Stefanidis D, Yonce TC, Green JM, Coker AP (2013) Cadavers versus pigs: which are better for procedural training of surgery residents outside the OR? Surgery 154(1):34–37. 10.1016/j.surg.2013.05.001 23809483 10.1016/j.surg.2013.05.001

[CR63] Stovall DW, Fernandez AS, Cohen SA (2006) Laparoscopy training in United States obstetric and gynecology residency programs. JSLS 10(1):11–15 16709349 PMC3015675

[CR64] Supe A, Dalvi A, Prabhu R, Kantharia C, Bhuiyan P. Cadaver as a model for laparoscopic training. Indian J Gastroenterol. 2005;24(3):111–113. 16041103

[CR65] Sutton ERH, Billeter A, Druen D, Roberts H, Rice J (2017) Development of a human cadaver model for training in laparoscopic donor nephrectomy. Clin Transpl. 10.1111/ctr.12979 10.1111/ctr.12979 28342285

[CR66] Tamate M, Matsuura M, Kanao H, Saito T (2022) A mixed-method evaluation of cadaver surgery training for gynecologic oncology. J Obstet Gynaecol Res 48(12):3252–3261. 10.1111/jog.15424 36128608 10.1111/jog.15424

[CR67] Tjalma WA, Degueldre M, Van Herendael B, D’Herde K, Weyers S (2013) Postgraduate cadaver surgery: an educational course which aims at improving surgical skills. Facts Views vis Obgyn 5(1):61–65 24753929 PMC3987353

[CR68] Udomsawaengsup S, Pattana-arun J, Tansatit T et al (2005) Minimally invasive surgery training in soft cadaver (MIST-SC). J Med Assoc Thai 88 Suppl 4:S189-S194.ü 16623027

[CR69] Usami T, Fujioka T, Yoshida A et al (2018) Assessment of laparoscopic training for gynecological malignancies using Thiel-embalmed human cadavers. Mol Clin Oncol 9(5):511–514. 10.3892/mco.2018.1715 30345045 10.3892/mco.2018.1715 PMC6174390

[CR70] Vlaovic PD, Sargent ER, Boker JR et al (2008) Immediate impact of an intensive one-week laparoscopy training program on laparoscopic skills among postgraduate urologists. JSLS 12(1):1–8 18402731 PMC3016039

[CR71] Wedel T, Ackermann J, Hagedorn H, Mettler L, Maass N, Alkatout I (2019) Educational training in laparoscopic gynecological surgery based on ethanol-glycerol-lysoformin-preserved body donors. Ann Anat 221:157–164. 10.1016/j.aanat.2018.10.002 30312766 10.1016/j.aanat.2018.10.002

[CR72] White SA, Satchidanand RY, French JJ, Tait IZ, Manas DM (2014) A cadaver lab training facility to facilitate laparoscopic liver resection. Surg Laparosc Endosc Percutan Tech 24(4):357–360. 10.1097/SLE.0000000000000046 24752163 10.1097/SLE.0000000000000046

[CR73] Wyles SM, Miskovic D, Ni Z et al (2011) Analysis of laboratory-based laparoscopic colorectal surgery workshops within the English National Training Programme. Surg Endosc 25(5):1559–1566. 10.1007/s00464-010-1434-y 21058021 10.1007/s00464-010-1434-y

[CR74] Yiasemidou M, Roberts D, Glassman D, Tomlinson J, Biyani S, Miskovic D (2017) A multispecialty evaluation of Thiel cadavers for surgical training. World J Surg 41(5):1201–1207. 10.1007/s00268-016-3868-4 28144746 10.1007/s00268-016-3868-4 PMC5394144

[CR75] Yoshida S, Miyano G, Tanaka M et al (2021) Cadaver training for minimally invasive pediatric surgery: a preliminary report. J Laparoendosc Adv Surg Tech A 31(12):1485–1490. 10.1089/lap.2021.0333 34846942 10.1089/lap.2021.0333

[CR76] Zevin B, Aggarwal R, Grantcharov TP (2012) Simulation-based training and learning curves in laparoscopic Roux-en-Y gastric bypass. Br J Surg 99(7):887–895. 10.1002/bjs.8748 22511220 10.1002/bjs.8748

