# Correction: A systematic review of the use of human body donor models for postgraduate laparoscopic surgical training

**DOI:** 10.1007/s12565-025-00889-4

**Published:** 2025-08-04

**Authors:** Sedat Alp Pinar, Joseph J. Morrow, Chia Yew Kong

**Affiliations:** Academic Unit of Surgery, Glasgow Royal Infirmary and University of Glasgow, New Lister Building, Glasgow, G31 2ER UK

**Correction: Anatomical Science International** 10.1007/s12565-025-00872-z

In Fig. 1 of this article, the records from other sources should be n = 11 instead of n = 8; the figure should have appeared as shown below. In the sentence beginning ‘Rai et al. (2015) used TEBDs for...’ in this article, the reference citation ‘Rai et al. (2015)’ should have read ‘Rai et al. (2012)’.

**Fig. 1:**
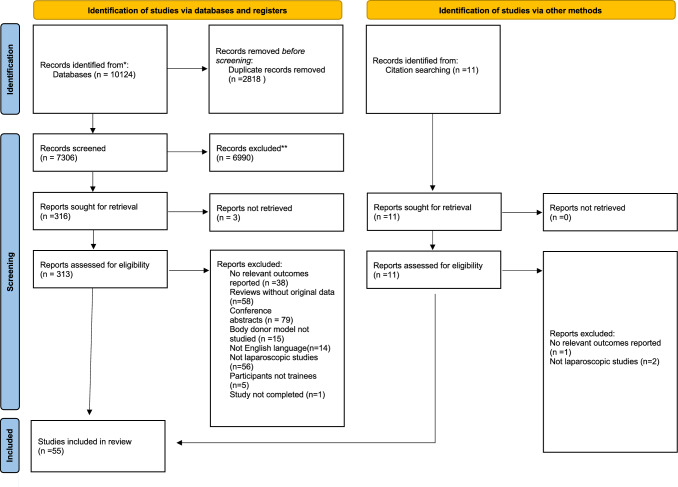
PRISMA flow diagram of the literature search and inclusion process

The original article has been corrected.

