# Supplementary material for: A systematic review of the use of human body donor models for postgraduate laparoscopic surgical training
Source: Anat Sci Int. 2025 Jul 17;101(3):241–67. doi: 10.1007/s12565-025-00872-z (PMC13179167; doi:10.1007/s12565-025-00872-z)
Supplement: Supplementary file 1 — Supplementary file1 (DOCX 15 KB) [file 12565_2025_872_MOESM1_ESM.docx]

**Supplement**

| **Medline** | **Embase** | **Clinicaltrials.gov** | **Cochrane database** |
| --- | --- | --- | --- |
| 1. exp cadaver/ 2. cadaver.mp 3. body donor.mp 4. fresh frozen.mp 5. embalm*.mp 6. human cadaver.mp 7. exp Education, Medical 8. surg* education.mp 9. surg* training.mp 10. exp “internship and residency” 11. exp Education, Medical, Graduate/ 12. simulation.mp 13. 1 OR 2 OR 3 OR 4 OR 5 OR 6 14. 7 OR 8 OR 9 OR 10 OR 11 OR 12 OR 15. 13 AND 14 | 1. exp Cadaver/ 2. cadaver.mp 3. body donor.mp 4. fresh frozen.mp 5. embalm*.mp 6. human cadaver.mp 7. exp Education, Medical 8. surg* education.mp 9. surg* training.mp 10. exp “internship and residency”/ 11. exp Education, Medical, Graduate/ 12. simulation.mp 13. 1 OR 2 OR 3 OR 4 OR 5 OR 6 14. 7 OR 8 OR 9 OR 10 OR 11 OR 12 15. 13 AND 14 | Surgical simulation AND cadaver;  Surgical training AND cadaver;  Simulation AND cadaver | 1. surg* education 2. surg* simulation 3. cadaver 4. body donor 5. 1 OR 2 6. 3 OR 4 7. 5 AND 6 |

**Table 1. Search strategy used for our literature search on the 4 different databases.**
